# Assessment of the Safety Profile of Purified *Pentadesma butyracea (Clusiaceae)* Gum Intended as a Pharmaceutical Excipient

**DOI:** 10.1155/jt/4227246

**Published:** 2026-04-01

**Authors:** Mary-Ann Archer, Kwabena Ofori-Kwakye, Raphael Johnson, Ernest Amponsah Asiamah, Wisdom Ahlidja, Mustapha Abubakar Ahmed, Isaac Tabiri Henneh

**Affiliations:** ^1^ Department of Pharmaceutics, University of Cape Coast, Cape Coast, Ghana, ucc.edu.gh; ^2^ Department of Pharmaceutics, Kwame Nkrumah University of Science and Technology, Kumasi, Ghana, knust.edu.gh; ^3^ Department of Biomedical Sciences, University of Cape Coast, Cape Coast, Ghana, ucc.edu.gh; ^4^ Department of Pharmacotherapeutics and Pharmacy Practice, University of Cape Coast, Cape Coast, Ghana, ucc.edu.gh

**Keywords:** gum, *Pentadesma butyracea*, pharmaceutical excipients, toxicity profile

## Abstract

*Pentadesma butyracea* (family *Clusiaceae*) bark is widely used in traditional medicine across sub‐Saharan Africa. However, the investigation of the safety profile of the gum exudate obtained from the stem bark of the plant is undocumented. This study evaluated the acute and subacute toxicity of the purified *Pentadesma butyracea* gum (PBG) in 36 male Sprague Dawley rats (8–10 weeks old; 100–182.08 g) to determine its safety profile for potential use as a pharmaceutical excipient. Acute toxicity was assessed using a single oral dose of 2000 mg/kg PBG, while subacute toxicity involved daily administration of 250, 500, and 1000 mg/kg PBG for 28 days. In the acute toxicity study, no mortality or significant adverse effects on behavior, body weight, relative organ weight, or histological features were observed, suggesting an LD_50_ greater than 2000 mg/kg PBG. Hematological and biochemical analyses revealed no harmful deviations, supporting the safety of PBG in acute exposure. In the subacute study, no mortality occurred across all doses, and body weight changes were minimal. Relative organ weights of the kidneys, heart, and lungs increased at higher doses, indicating potential dose‐dependent effects. Biochemical analyses revealed no significant alterations in liver enzymes (AST, ALT, and ALP) or markers of kidney function (urea and creatinine) at lower doses; however, slight elevations in bilirubin and creatinine were observed at 1000 mg/kg PBG, suggesting mild hepatic and renal stress. Histopathological analysis confirmed the absence of severe pathological changes, with only mild and reversible alterations at higher doses. Overall, PBG demonstrated a favorable safety profile at doses below 1000 mg/kg, supporting its potential use as a pharmaceutical excipient. The preliminary phytochemical screening also showed the presence of tannins, glycosides, coumarins, sterols, and triterpenoids. Further studies, including chronic toxicity and pharmacokinetic assessments, are recommended to establish the long‐term safety of PBG.

## 1. Introduction

In Ghana and most developing countries, almost all pharmaceutical excipients used by local companies are synthetic and are mostly imported from South‐East Asia and developed countries. This huge financial investment (high cost of synthetic excipients, shipping, and import duties) contributes to the overall high cost of treatment due to the increased cost of overall medication. This defies Sustainable Development Goal 3 (SDG 3), which aims to prevent unnecessary suffering from avoidable diseases and premature death via the concentration on key targets that improve the health of a country’s population [1]. This could be achieved by the reduction of disease mortality through the provision of affordable, safer, and equally efficacious medicaments. One such approach is the development of pharmaceutical excipients from locally sourced natural materials. This would not only allow the population to get access to safe medicaments via the elimination of the huge cost of shipping and import duties but also ensure the safe production of medicaments. Thus, almost all active pharmaceutical ingredients (APIs) are chemically synthesized and, as such, have been found to possess mild to serious side effects. As a result, it becomes important that excipients used in their formulation are not only compatible with the API but also do not add onto the already established side effects of the API. Thus, the use of synthetic excipients also increases the occurrence of adverse drug effects of medications prepared from them. An example is using colorants, such as tartrazine, in pharmaceutical preparations, which causes headaches and asthma exacerbation. Amaranth is carcinogenic, and its use has been banned in some countries. Also, alcohols such as benzyl alcohol used as a preservative in topical preparations cause neurological effects, and when used as a stabilizer in parenteral preparations, they cause gasping syndrome in neonates, which is fatal [2]. As a result, there is an immediate need for Ghana and other developing countries to develop pharmaceutical excipients from locally sourced materials to aid in the eradication of this crux.

Commercially, gums are vital excipients in pharmaceutical formulations. Gums have evolved from their use as binders, disintegrants, suspending agents, and emulsifying agents to excipients used as novel drug delivery carriers in the design and development of matrix formulations, gene therapy, and nanomedicine [3–5]. Natural gums have been of interest to researchers compared to synthetic gums due to their perceived nontoxicity, bioavailability, low cost, and abundant availability [5, 6].

*Pentadesma butyracea*, belonging to the family Clusiaceae, is an evergreen tropical tree commonly found in the forest zones of West and Central Africa, stretching from Guinea‐Bissau to Cameroon and extending southward to Gabon and the Congo region [7]. This plant, known locally as “Paa” or “Agya Paa” in Ghana, has been extensively utilized in traditional medicine for its diverse therapeutic properties. Its bark, leaves, seeds, and roots have demonstrated antibacterial, antileishmanial, antiplasmodial, and antitumor activities [8–11]. Additionally, the plant’s butter, derived from its seeds, is used as massage oil and in cosmetic formulations for skincare, hair care, and soap softening due to its lubricating and healing properties [12]. Despite the prospects of purified *Pentadesma butyracea* gum (PBG) as a potential pharmaceutical excipient, there is a paucity of data on its acute and repeated dose toxicity.

Toxicity assessments, including acute and subacute studies, are fundamental for determining the safety profile of medicinal plants and pharmaceutical excipients. Acute toxicity tests evaluate the immediate harmful effects following a single high‐dose administration, while subacute toxicity studies assess the effects of repeated exposure over weeks [13, 14]. Such evaluations provide critical information on organ‐specific toxicities, hematological alterations, biochemical changes, and histopathological impacts, ensuring the safety and efficacy of plant‐derived therapeutics [15, 16].

The present study aims to evaluate the acute and subacute toxicity of purified PBG in male Sprague Dawley rats. By analyzing behavioral, hematological, biochemical, and histopathological parameters, this study seeks to establish a comprehensive safety profile for purified PBG, thereby supporting its potential as a safe pharmaceutical excipient.

## 2. Method

### 2.1. Collection, Extraction, and Purification of PBG

The dried gum was collected from the incised stem bark of *Pentadesma butyracea* plants found within the Nkoranza North municipality (7° 43′ 8.7″N, 1° 40′ 43.9″W) in the Bono East region of Ghana. It was authenticated by the botanist at the Department of Herbal Medicine, Kwame Nkrumah University of Science and Technology (KNUST), Kumasi. A voucher specimen with authentication number KNUST/HM1/2025/G001 was subsequently deposited in the Herbarium Unit of the Department of Herbal Medicine. Unwanted materials such as dust, stones, bark, and soil were removed from the collected gum while cleaning it. The extraction and purification of the gum were carried out via modification of an earlier method [17]. A quantity of 200 g of crude milled (Retsch Mill SK‐1) PBG was weighed into a container. A volume of 1.8 L of distilled water was added and allowed to stand for 24 h for the gum to go into solution. It was then passed through a sieve with a mesh size of 90 μm. The filtrate was again filtered using a calico. A volume of 1.8 L of absolute ethanol was used to precipitate the purified gum from the filtrate. The precipitate was then dried in an electric oven at 50°C for 24 h. The dried purified gum was then blended using a grinder (Nima Japan Grinder, China). It was then sieved through a mesh of size 125 μm. The gum was stored in a ziplock until needed for further analysis.

### 2.2. Phytochemical Screening of Purified PBG

#### 2.2.1. Test for Glycosides

Two hundred milligrams of gum was warmed in 5 mL of dilute HCl in a water bath for 2 min. The mixture was filtered, and 3 drops of 20% NaOH were added to the filtrate to make it alkaline, which was confirmed by using litmus paper (which turned red litmus paper blue). One milliliter of Fehling’s solutions A and B was added and heated on a water bath for 2 min. The formation of a brick‐red precipitate indicated the presence of glycosides [18].

#### 2.2.2. Test of Tannins

A quantity of 0.4 g of gum was boiled in 20 mL of water for 5 min. The mixture was cooled and filtered. The volume of the filtrate was adjusted to 20 mL with distilled water. A volume of 3 mL of 1% gelatin solution was added to 3 mL of the diluted filtrate. The formation of a white gelatinous precipitate indicated the presence of tannins [18].

#### 2.2.3. Test for Alkaloids

Four hundred milligrams of gum was boiled in 20 mL ammoniacal alcohol (1 part of strong ammonia:9 parts of 95% alcohol). It was then cooled and filtered. The filtrate was evaporated to dryness. The residue was extracted with 5 mL of 1% H_2_SO_4_. The mixture was filtered. Five milliliters of ammonia solution was added to the filtrate to make it alkaline. The alkalinity of the mixture was confirmed using litmus paper. A volume of 5 mL of chloroform was added to the filtrate and shaken. A volume of 2.5 mL of the chloroformic layer was evaporated to get rid of the chloroform. The resulting residue was dissolved in 1% H_2_SO_4_, and a drop of Dragendorff’s reagent was added to it. The formation of an orange‐red precipitate indicated the presence of alkaloids [18].

#### 2.2.4. Test for Flavonoids

A quantity of 200 mg of gum was dissolved in 5 mL of water, and the mixture was filtered. A paper was dipped into the filtrate and allowed to dry. The dried paper was exposed to a concentrated ammonia solution and then to HCl. The appearance of an intense yellow color, which disappears on exposure to HCl, indicated the presence of flavonoids [18].

#### 2.2.5. Test for Coumarins

Two hundred milligrams of gum was dissolved in 10 mL of chloroform and filtered. Five milliliters of the filtrate was evaporated to dryness. The resulting residue was dissolved in 5 mL of hot distilled water. It was then cooled, and 0.5 mL of 10% ammonia solution was added to the extract. The occurrence of an intense bluish‐green fluorescence under UV light indicated the presence of coumarins [18].

#### 2.2.6. Test for Sterols

An amount of 200 mg of gum was added to 10 mL of chloroform and filtered. Two drops of acetic anhydride were added to 5 mL of the filtrate, and 2 drops of concentrated H_2_SO_4_ were also carefully added to the side of the test tube to form a reddish‐brown ring at the interface. The formation of a bluish color at the top of the reddish‐brown interface indicated the presence of sterols [18].

#### 2.2.7. Test for Triterpenoids

Two hundred milligrams of gum was added to 10 mL of chloroform and filtered. To 5 mL of the filtrate, 2 drops of concentrated sulfuric acid were carefully added at the side of the tube to form a lower layer. The formation of a reddish‐brown at the interface indicated the presence of a triterpenoid nucleus [18].

#### 2.2.8. Experimental Animals

Thirty‐six male Sprague Dawley rats of 8–10 weeks old (100–182.08 g) used for this study were obtained from the Noguchi Memorial Institute for Medical Research (NMIMR), Ghana, and kept at the School of Biological Sciences’ animal house, University of Cape Coast (UCC). They were acclimatized for 14 days before the experiments under standard conditions of room temperature (25 ± 1°C) and relative humidity (40 ± 10%) with a 12/12 h light/dark cycle. The animals were fed with standard commercial pelleted rodent feed (Floor Mills of Ghana Limited, Tema, Ghana) and had access to clean water throughout the experiment. The National Research Council’s (2010) “Guide for Care and Use of Laboratory Animals” protocols were adhered to throughout the study. Approval for the study was granted by the Research and Ethics Committee of the School of Pharmacy and Pharmaceutical Sciences, University of Cape Coast, under certification number UCCSoPPS/REC/22/0013.

#### 2.2.9. Acute Toxicity

The acute toxicity study was conducted by following the Organization for Economic Co‐operation and Development Guidelines for Testing Chemicals 420, with slight adjustments [13]. Twelve healthy Sprague Dawley male rats were randomly assigned to two groups (*n* = 6/group). Although female rats are preferred in this test, male rats were used in this test to minimize variability associated with the estrous cycle, which can influence biochemical, hormonal, and toxicokinetic parameters and potentially confound interpretation of treatment‐related effects in keeping with other published studies [19, 20]. The adequacy of the sample size was determined by using the “resource equation” method [21]. According to this method, a value denoted as “E” is measured, representing the degrees of freedom in the analysis of variance (ANOVA). The value of E is expected to fall within the range of 10–20. It is calculated using the following formula:

(1) E=total number of animals−total number of groups. Thus, E=12210−=. This falls within the acceptable range.

The maximum recommended oral dose of 2000 mg/kg of PBG was administered to six rats in Group A, and distilled water (10 mL/kg, p.o.) was administered to the six rats in Group B. The rats were closely monitored for signs of toxicity, including changes in skin, fur, eyes, respiration, movement, and mucous membranes, at various intervals postextract administration and daily for up to 14 days. Their food and water intake were recorded daily, and their body weights were measured on Days 0, 7, and 14. On the 15^th^ day, the rats were anesthetized with 2%–3% isoflurane diluted in oxygen [22], and blood samples were collected via cardiac puncture for hematological and biochemical analyses. Animals were then humanely euthanized by cervical dislocation, and vital organs/tissues, such as the liver, heart, and kidney, were collected for histopathological examination. The carcasses of the animals were disposed of under local institutional ethical guidelines and the “Guidelines for the Euthanasia of Animals” published by the American Veterinary Medical Association [23].

### 2.3. Hematological Assay

Hematological parameters such as the total number of red blood cells (RBCs), the total count of white blood cells (WBCs), levels of hemoglobin (HGB), packed cell volume (PCV), hematocrit (HCT), platelet counts , mean cell hemoglobin concentration (MCHC), mean cell volume (MCV), and mean corpuscular hemoglobin (MCH) were assessed usingthe autohaematology analyser (URIT‐5250Vet, China).

### 2.4. Biochemical Analysis

After clotting for 30 min, blood samples were processed for serum by centrifugation at 3000 rpm for 5 min using the HUMAX‐K centrifuge from Human, Germany. The resulting sera were stored at −20°C until further use. Serum samples were then analyzed for various biochemical markers, including total protein, albumin, total bilirubin, alkaline phosphatase (ALP), alanine aminotransferase (ALT), and aspartate aminotransferase (AST) using the Selectra Junior Autoanalyzer (Vital Scientific Bv, Version 04, Netherlands).

### 2.5. Histopathology

The organs of the rats were examined for gross changes and weighed prior to tissue processing. Tissue sections from the heart, liver, lung, kidney, brain, and spleen were fixed in 10% phosphate‐buffered formalin for 24 h. The tissues were dehydrated with graded concentrations of ethanol and embedded in paraffin wax. A rotary microtome (Epredia HM 340E, Thermofisher Scientific, USA) was used to section the tissues (5 μm). The sectioned tissues were processed and stained with hematoxylin and eosin. The slides of the tissues were observed using a binocular light microscope with a digital camera (Olympus, Japan) connected to a computer using AmScope (MD500, USA). The micrographs of the tissues were examined for any pathological alterations .

### 2.6. Subacute Toxicity

The rats were randomly divided into four groups (*n* = 6/group). The sample size was determined using the “resource equation” method, as described under the acute toxicity section. Here, *E* = 24‐4 = 20. This falls within the acceptable range of 10–20 [21]. During analysis of data, four animals were randomly selected from each group and this sample size also falls within the acceptable range. Group A received distilled water, whereas Groups B, C, and D were orally treated with PBG at doses of 250 mg/kg, 500 mg/kg, and 1000 mg/kg, respectively, for 28 days via the gavage method. The rats were observed twice daily for morbidity and mortality throughout the study period. All animals had free access to food and water. They were weighed on Days 0, 7, 14, and 21. On the 29^th^ day, the rats were anesthetized with 2%–3% isoflurane diluted in oxygen [22]. Four animals from each group were then humanely euthanized by cervical dislocation, and blood samples were collected into EDTA and gel separator tubes for hematological and biochemical analyses, respectively, as described in the acute toxicity section. The vital organs, including the liver, heart, spleen, kidneys, and lungs, were removed, weighed, and evaluated histologically for any pathological changes as previously described. The carcasses of the animals were disposed of in accordance with local institutional ethical guidelines and the “Guidelines for the Euthanasia of Animals” published by the American Veterinary Medical Association [23]. The relative organ weight of each rat was calculated using the following formula:

(2) Relative organ weight=organ weight gbody weight of rat g×100.

### 2.7. Statistical Analysis

Data were analyzed using GraphPad Prism, Version 8.0 (GraphPad Software, San Diego, CA, USA) for Windows 10. Values obtained were presented as mean ± standard error of the mean (SEM) and considered statistically significant if *p* < 0.05. The dataset was assessed for normality using the Shapiro–Wilk test, and Levene’s test was applied to evaluate the homogeneity of variances. Following confirmation that the assumptions for normality and homogeneity of variances were met, one‐way ANOVA followed by Dunnett’s multiple comparison tests was used to determine differences between more than two groups. Two groups were compared using Student’s *t*‐test.

## 3. Results

### 3.1. Preliminary Phytochemical Screening

Results presented in Table 1 show that the purified PBG contained phytochemicals such as tannins, glycosides, sterols, coumarins, and triterpenoids. Saponins, flavonoids, and alkaloids were absent.

**TABLE 1:** Secondary metabolites tested in purified *Pentadesma butyracea* gum.

Phytochemical constituents	Observation	Inference
Tannins	Formation of a white gelatinous precipitate	Present
Glycosides	Formation of brick‐red precipitates	Present
Saponins	Absence of froth formation	Absent
Sterols	Formation of a bluish color at the top of the reddish‐brown interface	Present
Coumarins	Occurrence of an intense bluish‐green fluorescence under UV light	Present
Flavonoids	No appearance of an intense yellow color	Absent
Alkaloids	No formation of orange‐red precipitates	Absent
Triterpenoids	Formation of reddish‐brown color at the interface	Present

### 3.2. Acute Toxicity of Purified PBG

Acute toxicity testing during the 14 days did not show any significant change in the body weight of animals treated with 2000 mg/kg PBG compared to the control group (Table 2). Relative internal organ weight assessment revealed no statistically significant differences between the naïve control and PBG (2000 mg/kg) group after 14 days of acute toxicity testing (Table 3). Furthermore, the hematological and biochemical assessment did not differ between the 2000 mg/kg PBG compared to the control group (Tables 4 and 5).

**TABLE 2:** Weekly body weights (*g*) of animals in control and PBG groups after 14 days of acute toxicity testing.

Days	Control	2000 mg/kg
0	125.0 ± 11.03	139.0 ± 7.33
7	128.9 ± 11.94	142.2 ± 6.85
14	132.8 ± 6.94	145.9 ± 7.47

*Note:* Values are expressed as mean ± SEM (*n* = 6). *p* > 0.05, compared with the control group using Student′s *t*‐test.

**TABLE 3:** Relative internal organ weight (*g*) of rats given PBG (2000 mg/kg) group after 14 days of acute toxicity testing.

Organs	Control	2000 mg/kg
Left kidney	0.3190 ± 0.021	0.3883 ± 0.015
Right Kidney	0.3123 ± 0.028	0.3615 ± 0.018
Liver	2.904 ± 0.269	3.068 ± 0.154
Heart	0.3788 ± 0.036	0.4300 ± 0.032
Lungs	0.6240 ± 0.049	0.8875 ± 0.221
Spleen	0.2095 ± 0.011	0.2290 ± 0.016

*Note:* Values are expressed as mean ± SEM (*n* = 6). *p* > 0.05, compared with the control group using Student′s *t*‐test.

**TABLE 4:** Hematological profile of rats administered with PBG (2000 mg/kg) after 14 days of acute toxicity testing.

Hematological parameters	Control	2000 mg/kg
WBC (10^9^/L)	4.618 ± 1.360	2.863 ± 0.581
RBC (10^12^/L	7.503 ± 0.286	4.417 ± 1.496
Hemoglobin (g/dL)	14.45 ± 0.226	11.52 ± 1.423
HCT %	45.32 ± 2.483	42.58 ± 1.074
MCV (fL)	60.58 ± 3.046	55.38 ± 0.737
MCH (pg)	19.42 ± 0.971	36.90 ± 11.790
MCHC (g/dL)	32.43 ± 2.027	26.97 ± 4.299
Platelet (10^9^/L)	754.2 ± 135.0	1090 ± 292.3

*Note:* Values are expressed as mean ± SEM (*n* = 6). *p* > 0.05, compared with the control group using Student′s *t*‐test.

**TABLE 5:** Biochemical profile of rats administered with PBG (2000 mg/kg) for 14 days after acute toxicity testing.

Biochemical parameters	Control	2000 mg/kg
Direct bilirubin (umol/L)	2.483 ± 0.210	2.860 ± 0.350
Total bilirubin (umol/L)	9.117 ± 1.434	9.300 ± 0.730
Total protein (g/lL	87.48 ± 3.804	82.58 ± 6.014
Albumin (g/L)	56.10 ± 5.381	36.50 ± 5.495
AST (U/L)	111.8 ± 5.822	106.6 ± 11.86
ALT (U/L)	98.17 ± 9.506	82.20 ± 7.599
ALP (U/L)	718.0 ± 72.56	502.4 ± 97.33
GGT (U/L)	12.33 ± 2.552	12.60 ± 4.179
Urea (mmol/L)	8.182 ± 1.295	5.82 ± 1.048
Creatinine (mmol/L)	107.7 ± 10.94	97.60 ± 25.04
Globulin (g/L)	39.22 ± 14.49	46.08 ± 6.36

*Note:* Values are expressed as mean ± SEM (*n* = 6). *p* > 0.05, compared with the control group using Student′s *t*‐test.

### 3.3. Histological Examination of Rats’ Vital Organs in the Acute Toxicity Study of Purified PBG

**FIGURE 1:**
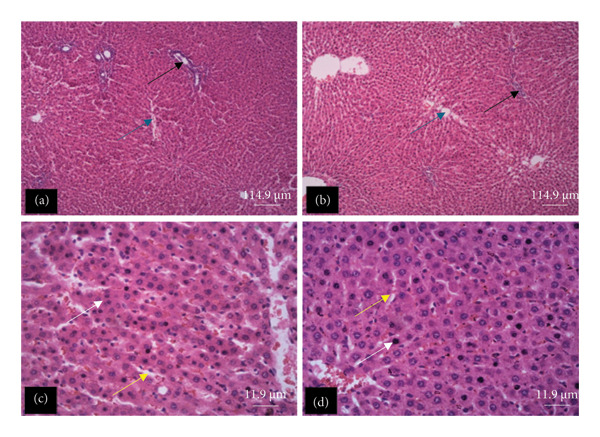
Hematoxylin and eosin‐stained sections of the cerebral cortex of rats treated with PBG and control for 14 days (a–d); (a) and (b) control group; (c) and (d) PBG‐treated rats. The yellow arrow represents the outer granular layer of neurons. The black arrow represents pyramidal neurons. Scale bars = 114.9 and 11.9 µm, respectively, for (a‐b) and (c‐d).

**FIGURE 2:**
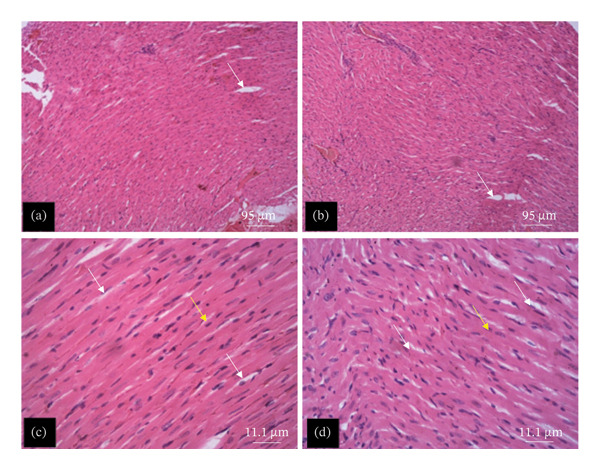
Hematoxylin and eosin–stained sections of the heart of rats treated with PBG and control for 14 days; (a) and (b) control group; (c) and (d) PBG‐treated rats. The white arrow represents sinusoids; the yellow arrow represents cardiomyocytes. Scale bars = 95 and 11.1 µm, respectively, for (a‐b) and (c‐d).

**FIGURE 3:**
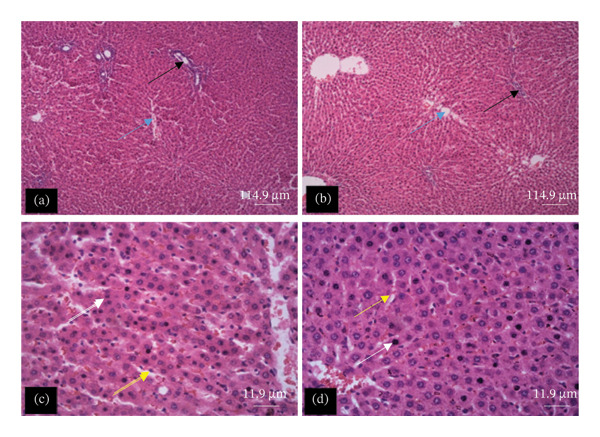
Hematoxylin and eosin–stained sections of liver of rats treated with PBG and control for 14 days (a–d); (a) and (b) control group; (c) and (d): PG‐treated rats. The blue arrow represents the central vein, the black arrow represents portal triad structures, the yellow arrow represents sinusoids, and the white arrow represents hepatocytes. Scale bars = 114.9 and 11.9 µm, respectively, for (a‐b) and (c‐d).

**FIGURE 4:**
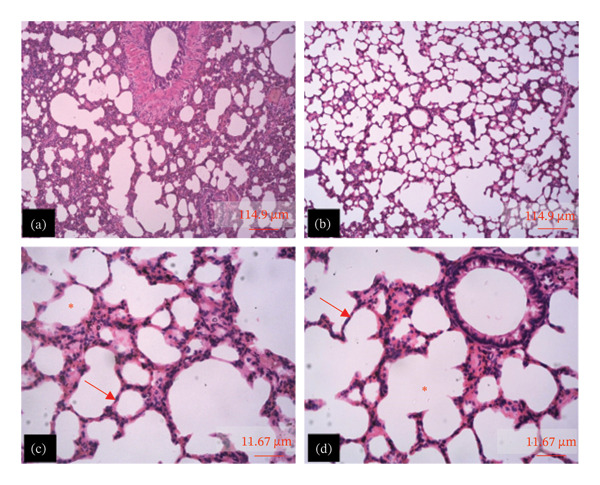
Hematoxylin and eosin–stained sections of the lung of rats treated with PBG and control for 14 days; (a) and (b) control group; (c) and (d) PBG‐treated rats. The red arrow represents the alveolar septa; the red asterisk represents the alveolus. Scale bars = 114.9 and 11.67 µm, respectively, for (a‐b) and (c‐d).

**FIGURE 5:**
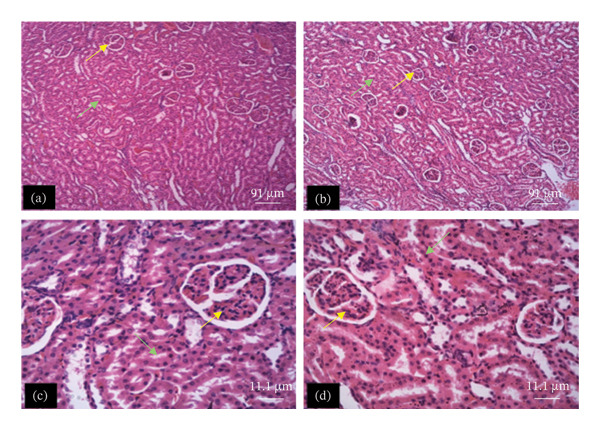
Hematoxylin and eosin–stained sections of kidney of rats treated with PBG and control for 14 days; (a) and (b) control group; (c) and (d) PBG‐treated rats. The yellow arrow represents renal corpuscles; the green arrow represents renal tubules/tubulointerstitium. Scale bars = 91 and 11.1 µm, respectively, for (a‐b) and (c‐d).

**FIGURE 6:**
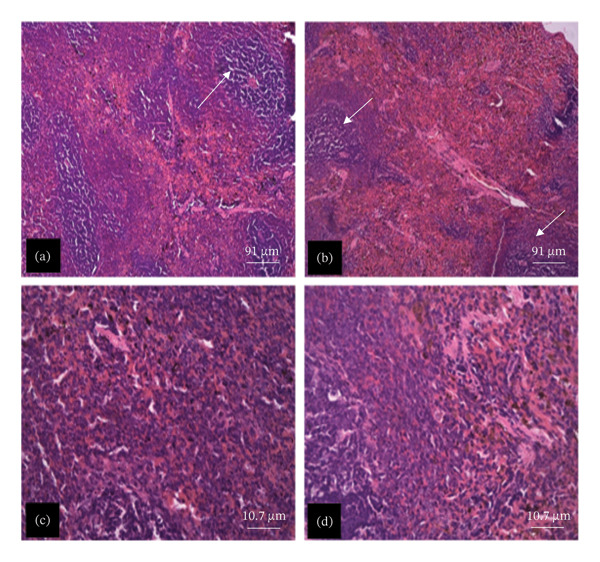
Hematoxylin and eosin–stained sections of spleen of rats treated with PBG and control for 14 days; (a) and (b) control group; (c) and (d) PBG‐treated rats. The white arrow represents white pulp; the yellow arrow represents the red pulp. Scale bars = 91 and 10.7 µm, respectively, for (a‐b) and (c‐d).

**FIGURE 7:**
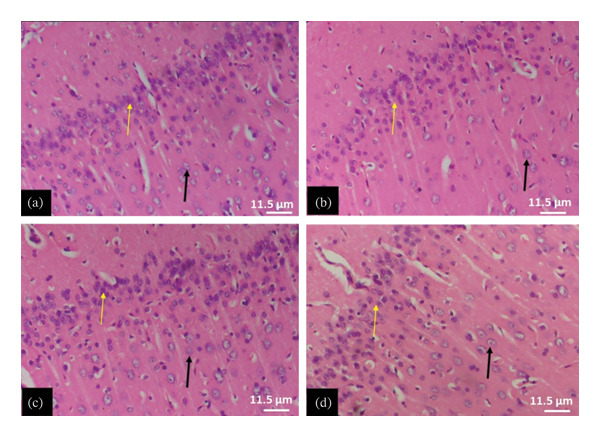
The photomicrograph of the cerebral cortex of rats treated with PBG and control for 28 days H&E stain; (a) control; (b) 250 mg/kg PBG; (c) 500 mg/kg PBG; (d) 1000 mg/kg PBG. The yellow arrow represents the outer granular layer of neurons. The black arrow represents pyramidal neurons. Scale bar = 11.5 µm.

The tissue micrographs (Figures 1, 2, 3, 4, 5, 6, 7) illustrate the effect of 14 days of PBG administration on various organs of rats. The cerebral cortex histoarchitecture in the PBG‐treated group (C and D), compared to the control (A and B), exhibits neuronal cell bodies of similar sizes and no infiltration of oligodendrocytes in the molecular layer (Figure 1). In Figure 2, the histoarchitecture of the heart in the PBG‐treated group (g and h), as opposed to the control, shows comparable sinusoids, normal cardiomyocytes, and an absence of inflammatory cells and fibrosis. The liver histoarchitecture in the PBG‐treated group (Figures 3, 3(c), and 3(b)), in contrast to the control, reveals no central vein congestion, intact portal triad structures with no evident fibrosis, and normal sinusoids without significant mononuclear lymphocytic infiltration. However, both periportal and centrilobular hepatocytes appear slightly hypertrophied with minimal pyknosis but maintain a normal appearance. Blood enters the liver bed at the portal triad area and flows through the sinusoids to the central vein. Periportal hepatocytes are initially at risk of injury; however, these cells display normal morphology (Figure 3). Figure 4 shows the lung histoarchitecture in the PBG‐treated group (a and b), compared to the control (c and d), which reveals similar sizes of alveolar sacs. The PBG‐treated group demonstrated no thickening of the basement membrane of the alveoli and no infiltration of inflammatory cells in the peribronchiolar, perivascular, or lung parenchyma. As presented in Figure 5, the kidney histoarchitecture in the PBG‐treated group (c and d), compared to the control, demonstrated normal renal corpuscles with no evident change in the size of the glomeruli and Bowman’s space. Additionally, there was no indication of lymphocytic infiltration. The tubulointerstitium remains unchanged, with no expansion in the peritubular spaces (indicative of hydropic change) or alterations in the size of proximal convoluted tubules (PCTs) or distal convoluted tubules (DCTs). In Figure 6, it was observed that the spleen histoarchitecture in the PBG‐treated group (a and b), compared to the control (c and d), exhibited no atresia of the white pulp region. Within the white pulp, the lymphoid follicles showed no activated germinal centers. The red pulp contains no karyorrhexic material, indicating minimal or no destruction of red blood cells.

### 3.4. Subacute Toxicity of Purified PBG

Treatment of animals with PBG (500 mg/kg and 1000 mg/kg) showed no difference in the weekly body weight measurement relative to the control group. However, there was a change in the body weight of animals administered with 250 mg/kg of PBG on the 14^th^ and 21^st^ days (Table 6). At 1000 mg/kg and 500 mg/kg of PBG, a statistically significant increase occurred in the kidney, heart, and lung (Table 7). The hematological analysis did not show any significant difference in the PBG‐treated group compared to the control group (Table 8). Table 9 showed elevated levels of total bilirubin in groups given 500 and 1000 mg/kg, respectively (^∗^ *p* < 0.05). However, there was a significant (*p* < 0.005) increase in creatinine levels in only the 250 mg/kg PBG dose.

**TABLE 6:** Weekly body weights (*g*) of animals given PBG (250, 500, 1000) mg/kg groups after 28 days of subacute toxicity testing.

Days	Control	250 mg/kg	500 mg/kg	1000 mg/kg
0	116.7 ± 5.419	147.9 ± 8.221	132.5 ± 7.422	126.8 ± 8.789
7	125.0 ± 6.562	157.6 ± 9.753	141.0 ± 6.673	135.1 ± 9.834
14	129.0 ± 7.037	164.0 ± 8.844^∗^	146.1 ± 6.092	142.0 ± 9.321
21	128.6 ± 9.791	168.5 ± 9.323^∗^	152.4 ± 5.805	148.4 ± 8.438
28	135.5 ± 9.771	172.8 ± 10.120	158.3 ± 6.228	154.1 ± 8.863

*Note:* Values are expressed as mean ± SEM (*n* = 6).

^∗^ *p* < 0.05 compared with the control group using one‐way ANOVA followed by Dunnett′s post hoc test.

**TABLE 7:** Relative internal organ weight of animals treated with PBG (250, 500, 1000) mg/kg group after 28 days of subacute toxicity testing.

Organs	Control	250 mg/kg	500 mg/kg	1000 mg/kg
Left kidney	0.3370 ± 0.004	0.3708 ± 0.005	0.3642 ± 0.022	0.4313 ± 0.024^∗^
Right kidney	0.3410 ± 0.003	0.3848 ± 0.006	0.3940 ± 0.009	0.4418 ± 0.026^∗∗^
Liver	3.781 ± 0.1385	4.237 ± 0.1913	4.170 ± 0.0518	4.396 ± 0.2717
Heart	0.3430 ± 0.003	0.3905 ± 0.009	0.4263 ± 0.016^∗^	0.4213 ± 0.027^∗^
Lungs	0.6038 ± 0.052	0.7008 ± 0.043	0.7830 ± 0.042	0.8323 ± 0.055^∗^
Spleen	0.2173 ± 0.016	0.2643 ± 0.014	0.2760 ± 0.011	0.2965 ± 0.028

*Note:* Values are expressed as mean ± SEM (*n* = 6).

^∗^ *p* < 0.05.

^∗∗^ *p* < 0.01 compared with the control group using one‐way ANOVA followed by Dunnett’s post *hoc* test.

**TABLE 8:** Hematological profile of rats administered with PBG (250, 500, 1000) mg/kg after 28 days of subacute toxicity testing.

Hematological parameters	Control	250 mg/kg	500 mg/kg	1000 mg/kg
WBC (10^9^/L)	4.570 ± 1.686	7.133 ± 1.143	6.482 ± 0.807	5.203 ± 0.535
RBC (10^12^/L)	7.175 ± 0.703	7.358 ± 0.184	7.368 ± 0.325	7.240 ± 0.504
Hemoglobin (g/dL)	13.08 ± 1.434	13.05 ± 0.463	13.14 ± 0.789	13.15 ± 1.024
HCT %	41.45 ± 6.304	44.93 ± 2.910	44.08 ± 3.235	43.50 ± 5.199
MCV (fL)	57.23 ± 4.405	61.18 ± 4.224	59.58 ± 1.794	59.58 ± 2.881
MCH (pg)	18.15 ± 0.239	18.55 ± 0.644	17.78 ± 0.549	18.13 ± 0.214
MCHC (g/dL)	32.25 ± 2.114	28.63 ± 2.877	29.92 ± 1.152	30.53 ± 1.195
Platelet (10^9^/L)	469.0 ± 162.7	883.0 ± 76.86	760.0 ± 53.51	850.8 ± 132.0

*Note:* Values are expressed as mean ± SEM (*n* = 6). *p* > 0.05 compared with the control group using one‐way ANOVA, thus indicating no statistical differences between control and treatment groups.

**TABLE 9:** Chemistry profile of rats administered with PBG (250, 500, 1000) mg/kg after 28 days of subacute toxicity testing.

Biochemical parameters	Control	250 mg/kg	500 mg/kg	1000 mg/kg
Direct bilirubin (umol/L)	3.550 ± 0.233	2.775 ± 0.214	3.400 ± 0.196	3.625 ± 0.165
Total bilirubin (umol/L)	9.650 ± 0.474	8.250 ± 0.731	12.18 ± 0.534^∗^	12.60 ± 0.265^∗^
Total protein (g/L)	90.35 ± 2.499	87.80 ± 7.635	83.63 ± 6.617	88.30 ± 11.61
Albumin (g/L)	41.15 ± 11.26	43.30 ± 7.95	32.53 ± 7.31	41.13 ± 6.02
AST (U/L)	96.50 ± 6.185	92.38 ± 5.482	99.25 ± 4.110	99.00 ± 3.082
ALT (U/L)	91.50 ± 3.175	95.75 ± 169	78.25 ± 6.486	90.25 ± 8.430
ALP (U/L)	746.0 ± 49.70	831.0 ± 16.21	792.5 ± 17.90	875.8 ± 91.05
GGT (U/L)	7.250 ± 2.287	5.250 ± 1.109	6.250 ± 1.493	11.25 ± 2.658
Urea (mmol/L)	4.04 ± 0.956	13.35 ± 2.609	4.94 ± 1.528	11.57 ± 3.258
Creatinine (mmol/L)	15.25 ± 3.497	165.5 ± 18.71^∗∗^	40.25 ± 8.340	85.00 ± 26.47
Globulin (g/L)	49.20 ± 12.91	44.78 ± 15.14	1.053 ± 0.31	78.80 ± 32.90

*Note:* Values are expressed as mean ± SEM (*n* = 6).

^∗^ *p* < 0.05.

^∗∗^ *p* < 0.01 compared with the control group using one‐way ANOVA followed by Dunnett’s post *hoc* test.

### 3.5. Histological Examination of 28‐Day Administration of Purified PBG

**FIGURE 8:**
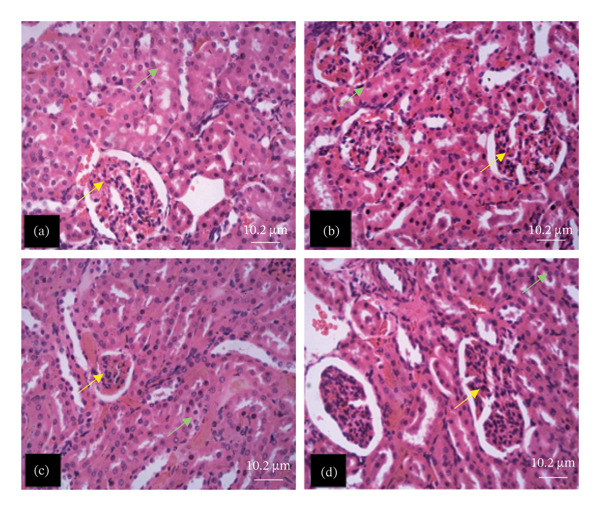
The photomicrograph of the kidney of rats treated with PBG and control for 28 days H&E stain; (a) control; (b) 250 mg/kg PBG; (c) 500 mg/kg PBG; (d) 1000 mg/kg PBG. The yellow arrow represents the glomerulus within renal corpuscles. The green arrow represents the proximal convoluted. Scale bar = 10.2 µm.

**FIGURE 9:**
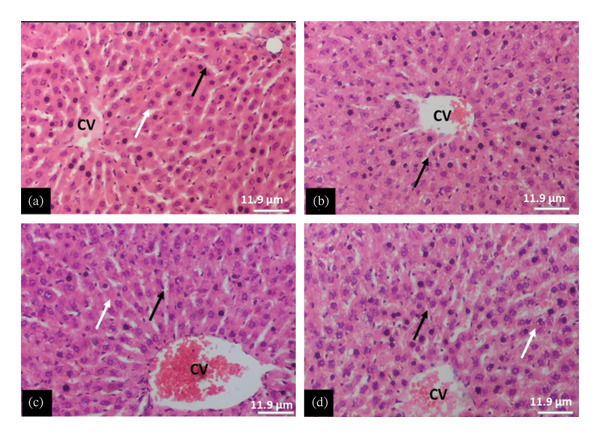
The photomicrograph of the liver of rats treated with PBG and control for 28 days H&E stain; (a) control; (b) 250 mg/kg PBG; (c) 500 mg/kg PBG; (d) 1000 mg/kg PBG. Scale bar = 11.9 µm.

**FIGURE 10:**
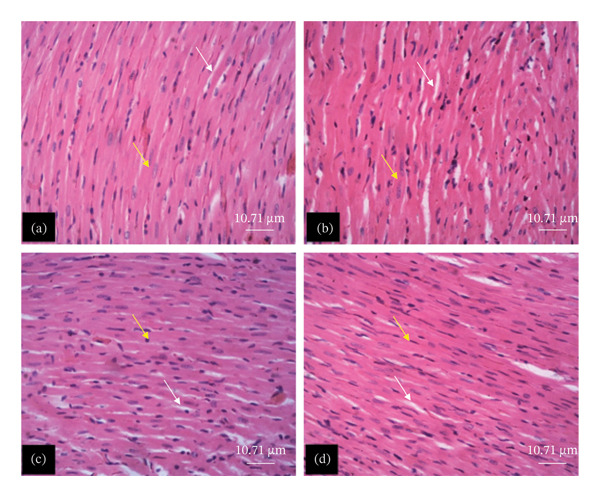
The photomicrograph of the heart of rats treated with PBG and control for 28 days H&E stain; (a) control; (b) 250 mg/kg PBG; (c) 500 mg/kg PBG; (d) 1000 mg/kg PBG. The white arrow represents sinusoids; the yellow arrow represents cardiomyocytes. Scale bar = 10.71 µm.

**FIGURE 11:**
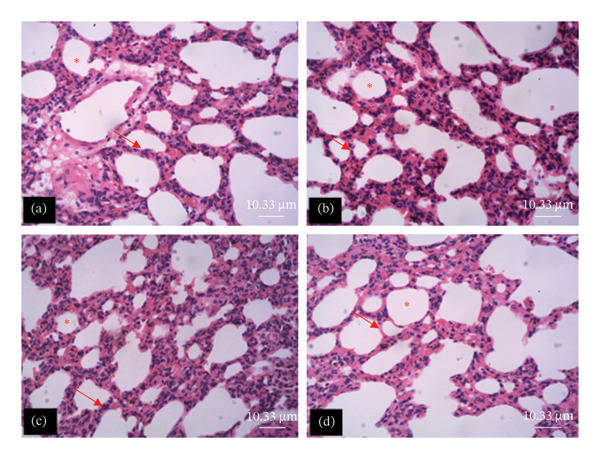
The photomicrograph of the lung of rats treated with PBG and control for 28 days H&E stain; (a) control; (b) 250 mg/kg PBG; (c) 500 mg/kg PBG; (d) 1000 mg/kg PBG. ^∗^Alveolus, and red arrow represents the alveolar wall. Scale bar = 10.33 µm.

**FIGURE 12:**
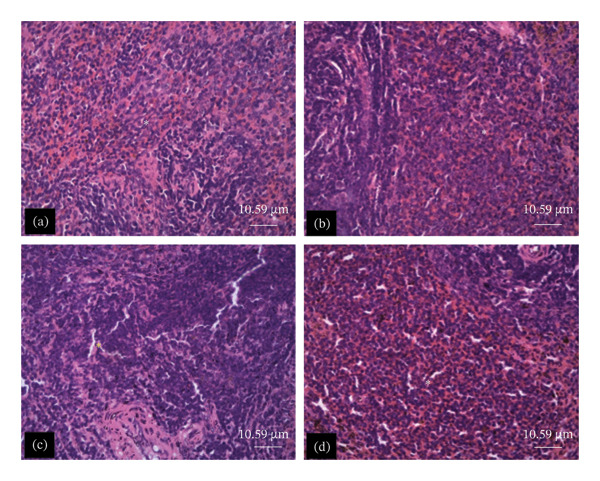
The photomicrograph of the spleen of rats treated with PBG and control for 28 days H&E stain; (a): control; (b): 250 mg/kg PBG; (c): 500 mg/kg PBG; (d): 1000 mg/kg PBG. White ∗ and yellow ∗ represent red pulp and white pulp. Scale bar = 10.59 µm.

Figures 7, 8, 9, 10, 11, 12 illustrate the histomorphological effect of a 28‐day PBG treatment on different organs of rats. Cerebral cortex histoarchitecture in the PBG‐treated group (b, c, and d), compared to the control, exhibited neuronal cell bodies of comparable sizes and no infiltration of oligodendrocytes in the molecular layer (Figure 7). The kidney histostructure (Figure 8) in PBG‐treated groups exhibited mild toxicity at the high doses, with no obvious pathological change at the lower doses. The low‐dose PBG‐treated group exhibited normal glomeruli and tubulointerstitium. Infiltration of mononuclear lymphocytic cells into the glomerulus at 1000 mg/kg, in addition to traces of pyknotic nuclei in the proximal convoluted tubules (PCTs), was also observed. From Figure 9, the liver histoarchitecture in the PBG‐treated group (c and d) exhibited no obvious fibrosis and normal sinusoids with no obvious mononuclear lymphocytic infiltration compared to the control (a). However, the hepatocytes exhibited minimal pyknosis yet appeared normal in all groups.

In Figure 10, the heart histoarchitecture in the PBG‐treated group (b, c, and d), compared to the control, exhibited no obvious changes in the myocardial fibers, no basement membrane thickening, no dilatation of the capillaries/sinusoids, and no infiltration of mononuclear lymphocytes.

Additionally, the lung microstructure appears normal in PBG‐treated groups (Figure 11). However, the lowest dose exhibited infiltration of mononuclear inflammatory cells, which was not observed in the higher doses (Figure 11). The images presented in Figure 12 show the histoarchitecture of the spleen. The red pulp of the lowest and highest dose PBG‐treated group exhibited normal histoarchitecture; the germinal centers were not activated, but increased infiltration of the mononuclear lymphocytes in the red pulp was observed. However, the middle dose exhibited expansion of the white pulp.

## 4. Discussion

The phytochemical screening of the gum demonstrated (Table 1) the presence of tannins, glycosides, sterols, coumarins, and triterpenoids but the absence of saponins, flavonoids, and alkaloids. This means that, when PBG is incorporated in any formulation, the antioxidative action of the tannins, sterols, and glycosides will stabilize the formulation via the scavenging of the free radicals present in the formulation. The tannins present also influenced the brownish color of the purified gum [24]. The antimicrobial activity of the glycosides and triterpenoids in the gum would ensure the microbial stability of the product [25–27]. Coumarins also possess phytoalexin properties. Thus, they also possess antimicrobial and antioxidant properties and hence would contribute to the overall stability of the formulation [28, 29]. These secondary metabolites also serve as medication, recreational drugs, flavoring, and aromatization agents [30], giving this gum additional vital benefits for its use as a pharmaceutical excipient.

The increasing demand for the use of natural gums in pharmaceutical preparations instead of their synthetic counterparts in recent times, as a result of their biocompatibility, purported safety, inertness, biodegradability, affordability, and readily availability, has necessitated toxicity assay of these excipients. This study evaluated the acute and subacute toxicity of purified PBG in male Sprague Dawley rats, aiming to establish its safety profile as a potential pharmaceutical excipient. These are established models according to the OECD for determining the safety of pharmaceutical products intended for use in humans [13, 31]. The findings indicated that PBG was well‐tolerated at lower doses, though dose‐dependent effects were observed at higher concentrations, emphasizing the importance of dose optimization for use in pharmaceutical preparations where a higher quantity of PBG per dose of medicament would be required for the formulation.

Mortality is considered a sensitive indicator in acute toxicity studies [32], and the absence of death at this high dose further underscores the gum’s safety. The acute toxicity study showed no mortality following a single oral administration of PBG at 2000 mg/kg, suggesting that the median lethal dose (LD_50_) of PBG exceeds this dose, and thus, the gum could be classified as a low‐toxicity substance according to OECD 420 guidelines [13]. The toxicity profile observed in the present study is consistent with previously published reports on *Pentadesma butyracea*, further supporting the relative safety of this plant. Earlier investigations of the hydroalcoholic extract of *P. butyracea* seeds demonstrated the absence of mortality and overt signs of toxicity in rodents following acute oral administration at doses up to 2000 mg/kg [10]. Similarly, oral administration of the stem bark decoction of *P. butyracea* did not induce acute toxic effects in Wistar rats at comparable doses, indicating a favorable safety margin [33].

In line with these findings, the current study showed no mortality or severe clinical signs of toxicity following administration of the test material, suggesting low acute toxicity. This observation aligns with studies reporting the absence of acute toxicity following exposure to hydroalcoholic extracts of *P. butyracea* leaves, as well as bark decoctions traditionally used in ethnomedicine [10, 33]. The lack of significant adverse effects may be attributed to the plant’s mild phytochemical profile, which is dominated by compounds with known biological compatibility and low toxic potential. Furthermore, studies investigating volatile constituents and other phytochemical fractions of *P. butyracea* have reported biological activities without associated toxic effects, reinforcing the notion that different plant parts share a comparable safety profile [34]. Collectively, these findings support the traditional use of *P. butyracea* bark, seeds, and leaves and suggest that the absence of acute toxicity observed in this study reflects an inherent low toxicological risk rather than a formulation‐specific effect.

In the 28‐day subacute toxicity study, repeated oral administration of PBG at doses of 250, 500, and 1000 mg/kg resulted in no significant mortality, reinforcing its safety. Body and relative organ weight changes, critical indicators of health, were comparable between treated and control groups, with minor fluctuations at higher doses. Alterations in relative organ weights of the kidney, heart, and lungs at higher doses were observed, indicating potential toxic effects that necessitate further examination [32]. These variations were explored in subsequent biochemical and histological analyses, providing additional insights.

Hematological analysis revealed no significant deviations in parameters such as RBC and WBC counts, HGB levels, HCT, and PCTs across all doses, indicating that PBG does not induce hematological toxicity [15, 32]. Biochemical evaluations showed no significant elevation in liver enzymes, including AST, ALT, and ALP, suggesting preserved hepatic function. These parameters can be used to monitor, detect, and characterize the harmful effects of toxic compounds as they provide crucial information for understanding the toxicological effects of test substances and their impact on several diseases [32, 35]. For instance, impaired or compromised hepatic function is characterized by elevated liver enzyme parameters such as AST, ALP, ALT, gamma‐glutamyl transferase (GGT), and total bilirubin levels. ALT is found in the cardiomyocytes and hepatocytes. Upon insult to the liver and the heart, the enzyme is released from the cytosol into the blood [36]. Similarly, ALP located in mucosal epithelia of the small intestine and proximal convoluted tubule of the kidney, bone, liver, and placenta is responsible for lipid transport in the intestine and calcification of bone. Another liver enzyme that is very critical in identifying liver disease is AST. There are two forms of AST, the mitochondrial and cytoplasmic forms. AST is abundant in the heart compared with the liver, skeletal muscle, and kidney [37]. It is imperative to mention that GGT is an enzyme of the hepatocyte and biliary epithelial cells, pancreas, and the intestine. It facilitates the transport of peptides across the membrane and glutathione metabolism. It is the only liver enzyme that is used to rule out bone disease as it is not found in bone [37]. In the present study, biochemical analysis of liver function markers, such as AST, ALT, and ALP, revealed no significant elevation in enzyme levels, suggesting preserved hepatic integrity. Moreover, elevated plasma proteins, albumin, and globulin, which are markers of hepatic injury, remained unchanged in the treatment groups. However, a dose‐dependent increase in total bilirubin at 1000 mg/kg suggests potential mild hepatic stress at higher doses [38].

Renal function test is used for assessing the function of the kidney. Renal function parameters such as serum creatinine and urea are traditional biomarkers for assessing the deleterious effect of toxicants on kidney function [35]. In the subacute toxicity study, renal function markers, such as urea and creatinine levels, were not significantly altered by PBG, but the low dose of PBG caused a significant increase in creatinine levels. The isolated increase in serum creatinine observed at 250 mg/kg, in the absence of similar changes at 500 and 1000 mg/kg, is likely attributable to a transient adaptive physiological response rather than dose‐related nephrotoxicity. Such patterns are well recognized in toxicological studies, particularly with phytochemical‐rich extracts, where low doses may elicit mild biochemical fluctuations while higher doses activate compensatory renal and metabolic mechanisms that normalize these parameters [39]. According to OECD Test Guideline 407, isolated biochemical alterations that lack dose dependency and are not supported by consistent changes in other renal markers or histopathological findings are generally considered nonadverse and biologically adaptive rather than toxicologically significant [14].

Histological evaluation of tissues or organs is an essential method used by physicians to corroborate physical and clinical diagnosis. The observed histopathological lesions, ranging from nonlethal changes including degeneration and congestion to fatal alterations like necrosis, suggest varying levels of toxic insult to the body’s organs. While degenerative changes typically suggest reversible, nonlethal cellular injuries, necrosis reflects irreversible, terminal cellular damage [40]. From these findings, it was observed that single‐dose administration of 2000 mg/kg of PBG had minimal effects on the organs, such as liver, kidney, brain, lungs, heart, and spleen, which suggest that the acute study is related to mild injury to the organs as shown in the hematological and biochemical analysis. On the other hand, the lungs exhibited infiltration of mononuclear inflammatory cells at a lower dose after 28‐day administration of PBG.

The observed safety of PBG is likely attributable to its phytochemical composition, which includes glycosides, triterpenoids, sterols, coumarins, and tannins, and other antioxidants known to mitigate oxidative stress and inflammation [11]. However, the mild hepatic and renal stress observed at higher doses underscores the dual role of these phytochemicals, which may confer therapeutic benefits at optimal doses but exhibit toxicity at excessive concentrations [37]. These findings highlight the need for further studies to elucidate the dose‐dependent mechanisms of toxicity and establish safe therapeutic ranges.

## 5. Conclusion

In conclusion, purified PBG was safe for acute and subacute use in rats at doses below 1000 mg/kg, with minimal adverse effects. These findings support its intended use as a pharmaceutical excipient. Nonetheless, further studies are essential to fully establish its long‐term safety. The findings of this study underscore the potential of purified PBG as a safe agent at appropriate doses. However, the dose‐dependent effects observed at higher concentrations warrant further studies, including chronic toxicity, mutagenicity, and carcinogenicity assessments, to comprehensively evaluate its safety profile. Additionally, detailed pharmacokinetic studies are recommended to understand the bioavailability and metabolism of its active constituents.

### 5.1. Limitations

In this study, only acute and subacute (28‐day) studies were conducted. Chronic toxicity and long‐term safety of PBG remain unassessed. Again, the study used only male rats, which excludes potential sex‐specific effects. Moreover, the study employed only a single animal species, which limits the generalizability of the findings. While the results are valuable for guiding future research, they may not be fully extrapolatable to humans or other animal models. Also, the absorption, distribution, metabolism, and excretion (ADME) profile of PBG was not evaluated, limiting understanding of how the gum behaves in vivo. Although mild hepatic and renal stress was noted at high doses, mechanistic studies (e.g., oxidative stress markers, inflammatory cytokines) were not conducted to clarify the underlying causes. Finally, while the total sample size of 36 rats was large enough, the sample size per group may be small, which can limit statistical robustness and increase the risk of Type II error.

## Author Contributions

Mary‐Ann Archer: conceptualization, methodology, formal analysis, investigation, and writing–draft manuscript. Kwabena Ofori‐Kwakye: conceptualization, supervision, and writing–review and editing. Raphael Johnson: conceptualization, supervision, and writing–review and editing. Ernest Amponsah Asiamah: formal analysis and methodology. Wisdom Ahlidja: methodology, investigation, and writing–review and editing. Mustapha Abubakar Ahmed: methodology and investigation. Isaac Tabiri Henneh: conceptualization, methodology, investigation, formal analysis, and writing–review and editing.

## Funding

No funding was received for this study.

## Ethics Statement

The approval of the study was granted by the Research and Ethics Committee of the School of Pharmacy and Pharmaceutical Sciences, University of Cape Coast, under certification number UCCSoPPS/REC/22/0013.

## Conflicts of Interest

The authors declare no conflicts of interest.

## Data Availability

The data that support the findings of this study are available from the corresponding author upon reasonable request.
